# Relationships between respiratory and oromotor events differ between motor phenotypes in patients with obstructive sleep apnea

**DOI:** 10.3389/fneur.2023.1150477

**Published:** 2023-03-21

**Authors:** Mutsumi Okura, Takafumi Kato, Midori Mashita, Hisae Muraki, Hideko Sugita, Motoharu Ohi, Mitsutaka Taniguchi

**Affiliations:** ^1^Department of Oral Physiology, Osaka University Graduate School of Dentistry, Suita, Osaka, Japan; ^2^Sleep Medical Center, Osaka Kaisei Hospital, Osaka, Japan; ^3^Center for Sleep Medicine, Asahi University Hospital, Gifu, Japan; ^4^Department of Psychiatry, Osaka University Graduate School of Medicine, Suita, Osaka, Japan

**Keywords:** obstructive sleep apnea, sleep bruxism, rhythmic masticatory muscle activity, arousal threshold, phenotype

## Abstract

**Purpose:**

The present study investigated the relationship between sleep bruxism (SB) and obstructive sleep apnea (OSA) in relation to the sleep architecture.

**Methods:**

We conducted a cross-sectional study. Polysomnographic recordings were performed on 36 patients. Sleep, respiratory, and oromotor variables, such as rhythmic masticatory muscle activity (RMMA) and non-specific masticatory muscle activity (NSMA), were compared between OSA patients with or without SB. A correlation analysis of the frequency of respiratory and oromotor events in NREM and REM sleep was performed. The frequency of oromotor events following respiratory events was also assessed.

**Results:**

The proportion of REM sleep was higher in OSA patients with SB than in those without SB (*p* = 0.02). The apnea-hypopnea index (AHI) did not significantly differ between the two groups; however, AHI was approximately 8-fold lower during REM sleep in OSA patients with SB (*p* = 0.01) and the arousal threshold was also lower (*p* = 0.04). Although the RMMA index was higher in OSA patients with than in those without SB (*p* < 0.01), the NSMA index did not significantly differ. The percentage of RMMA following respiratory events was significantly higher in OSA patients with than in those without SB, whereas that of NSMA did not significantly differ. The frequency of oromotor events throughout the whole night positively correlated with AHI. However, regardless of the sleep state, AHI did not correlate with the RMMA index, but positively correlated with the NSMA index.

**Conclusion:**

In consideration of the limitations of the present study, the results obtained indicate that OSA patients with SB have a unique phenotype of OSA and also emphasize the distinct relationship of respiratory events with RMMA and NSMA.

## 1. Introduction

Sleep bruxism (SB) is a masticatory muscle activity during sleep that is characterised as rhythmic (phasic) or non-rhythmic (tonic) and is not a movement disorder or a sleep disorder in otherwise healthy individuals (1). The comorbidity of sleep bruxism (SB) and obstructive sleep apnea (OSA) is an area of interest in research on sleep medicine and dentistry (2–5). The prevalence of SB in the adult population was previously reported to be 5.5–8% (6–8). OSA is more prevalent than SB, occurring in approximately 30% of the population (9). The prevalence of both SB and OSA is high, and SB increases the risk of negative oral health consequences (e.g., temporomandibular joint pain, prosthodontic complications), OSA is known to be associated with hypertension and various vascular diseases. It is very important to investigate the factors and effects of the combination of these two conditions. Epidemiological studies suggested that OSA patients are more likely to have SB (8), and this is supported by the findings of polysomnographic (PSG) studies showing that between 9 and 70% of OSA patients also have SB (10–14) and the body position-related OSA phenotype has higher SB and severe SB incidence (15). Genetic predisposition to OSA-SB relationship was also proposed previously (16).

Although studies have consistently reported the comorbidity of OSA and SB, their physiological relationship remains unclear. The idea that SB is a protective factor and may interrupt respiratory events is also a very concerned issue (17–19). Some studies found a positive correlation between the frequencies of respiratory and oromotor events (i.e., metrics of obstructive sleep apnea and sleep bruxism, respectively) during sleep (13, 20), whereas others did not (12). Other works showed the relationship SB and AHI in mild and moderate OSA, but not in severe OSA (11, 20). Even though a positive correlation was detected, its effects were moderate (11, 13). Moreover, only a weak temporal relationship was noted between rhythmic masticatory muscle activity (RMMA) and respiratory events (21), suggesting the lack of a direct causal relationship between respiratory and oromotor events. Two major reasons have been proposed for these discrepancies. Physiological factors, such as NREM sleep and REM sleep, that influence the occurrence of respiratory and oromotor events have not been well examined (22). Furthermore, motor activities related to respiratory events obscure the diagnosis of comorbid sleep-related movement disorders (i.e., periodic limb movement of sleep) and parasomnia (i.e., REM sleep behavior disorders) (23). In OSA patients, non-specific masticatory muscle activity (NSMA) was frequently detected following respiratory events regardless of the presence/absence of SB (17, 24–26). Therefore, motor phenotypes, such as RMMA and NSMA, may be significant confounding factors for interpreting the pathophysiological relationship between OSA and SB.

The present study investigated the characteristics of the sleep architecture, examined sleep state-dependent differences in respiratory and motor events, and analyzed the relationship between oromotor and respiratory events in OSA patients with SB.

## 2. Materials and methods

### 2.1. Subjects

Fifty-six outpatients (age: 51.4 ± 14.8 years old, F: 9, M: 47; BMI: 25.3 ± 5.3 kg/m^2^) were enrolled for the PSG evaluation of OSA at the Sleep Medical Center of Osaka Kaisei Hospital. They were interviewed by sleep physicians (MO, MO, and MT) and OSA was diagnosed based on a history of excessive daytime sleepiness, sleep disturbances, snoring, and witnessed respiratory pauses in sleep. Exclusion criteria were as follows: a history of neurological or psychiatric diseases; medical conditions, such as pulmonary, cardiac, and renal diseases; pregnancy; the use of medication such as analgesics and hypnotics. Prior to PSG recordings, subjects completed a written informed consent form approved by the Research Ethics Committee of Osaka University Graduate School of Dentistry, Osaka University Dental Hospital and Osaka Kaisei Hospital.

### 2.2. PSG recordings

Prior to PSG recordings, subjects completed a self-administered questionnaire on sleep disturbances and orofacial symptoms. They were interviewed on the subjective signs and symptoms of SB and OSA as well as on sleep quality. Intra- and extraoral examinations were performed to assess the clinical signs and symptoms of SB. PSG montages included the following biosignals: electroencephalograms (EEGs; C_3_M_2_, C_4_M_1_, O_1_M_2_, O_2_M_1_, F_3_M_2_, and F_4_M_1_); electro-oculograms (EOGs); electrocardiograms (ECGs); EMGs of the chin/suprahyoid, bilateral masticatory muscles (masseter and temporalis), and bilateral tibialis muscles; snoring sounds; nasal pressure and oronasal thermal airflow; chest and abdominal movements; arterial oxygen saturation; body position; and laryngeal movements. Audio and video recordings were simultaneously performed. All signals and audio videos were recorded using SomnoStar Pro (VIASYS, Yorba Linda, CA, United States) in Osaka Kaisei Hospital. Sleep recordings were initiated between 22:30 and 23:00 and ended between 06:30 and 07:30 or when subjects woke up.

### 2.3. Scores for sleep and oromotor variables

Sleep stages, arousals, and respiratory events were scored by a registered PSG technologist according to the AASM manual version 2.1 of the AASM criteria (27).

Respiratory events were scored as follows: apnea was defined as the cessation of airflow measured using oronasal thermal airflow lasting 10 s or more; hypopnea was defined as a decrease in nasal pressure airflow of >30% and a decline in SpO_2_ of >3% or as a decrease in nasal pressure airflow >30% associated with arousal. The frequency of respiratory events per hour of sleep was quantified as the apnea-hypopnea index (AHI). RMMA, including the phasic, mixed, and tonic types, and NSMA unrelated to RMMA were scored by investigators (MM and TK) using previously reported methods (17, 18, 27–29).

A low respiratory arousal threshold (ArTH), i.e., easy arousal from sleep in response to relatively mild airway obstruction, is one of the non-anatomical physiological factors. The relationship between SB and arousal has been well documented. In discussing the difference between OSA with and without SB, we thought that the classification of OSA phenotypes by ArTH might provide some clues to elucidate the pathophysiology. A low ArTH was defined as a score of two or higher on the following three-point scale: (AHI < 30) + (nadir SpO_2_ > 82.5%) + (fraction of hypopnea >58.3%). This approach has high sensitivity (80%) and specificity (88%) for the detection of OSA patients with a low ArTH. Additionally, the ArTH was calculated using AHI according to a recently validated score derived from standard PSG variables. We estimated the ArTH values of each subject using the following multiple linear regression model described by Edwards and colleagues: arousal threshold = −65.391 + (0.0636 × Age) + (3.692 × Sex [where male = 1, female = 0]) – (0.0314 × BMI) – (0.108 × AHI) + (0.533 × Nadir SpO_2_) + (0.0906 × % hypopnea) (30). The range proposed as significant for OSA pathogenesis are between 0 and − 15 cmH2O (30, 31).

### 2.4. Data analysis

The distribution of sleep stages was assessed for oromotor and respiratory events. AHI was also calculated for each sleep stage.

To assess temporal relationships between oromotor and respiratory events, the following oromotor events were analyzed. Oromotor events were scored when they occurred within 10 s of the end of respiratory events with or without respiratory event-related arousals. The percentages of these events in RMMA and NSMA were calculated.

The relationship between oromotor and respiratory events was also examined. The relationships between oromotor (sum of RMMA and NSNA), RMMA, and NSMA indices and respiratory events were investigated for the whole night, during NREM sleep, and during REM sleep. The relationships between the oromotor, RMMA, and NSMA indices and arousal events were also assessed for the whole night.

### 2.5. Statistical analysis

Descriptive statistics with means and standard deviations were used. Continuous variables with a normal distribution were analyzed by the *t*-test; otherwise, the Mann–Whitney U test was used. Frequency data in demographics were compared by Fisher’s exact test. Spearman’s rank correlation coefficient was used to examine the relationships between oromotor and respiratory events and arousal. All analyses were performed using EZR (Saitama Medical Center, Jichi Medical University, Saitama, Japan) (32).

## 3. Results

Among 56 subjects, 11 being treated by C-PAP and 5 with missing data were excluded. Subjects diagnosed with OSA based on PSG research diagnostic criteria (AHI ≥5 times/h) and no other sleep related disease were included in this study. Thirty-six subjects (male: 29, female: 7, mean age: 49.6 ± 14.2 years, BMI: 24.5 ± 3.4 kg/m^2^) were included and divided into two groups: 26 (male: 20, female: 6, mean age: 49.7 ± 14.1 years, BMI: 24.3 ± 3.8 kg/m^2^, AHI: 29.4 ± 23.6/h) into the OSA group (RMMA index <2 times/h) and 10 (male: 9, female: 1, mean age: 49.3 ± 15.3 years, BMI: 25.0 ± 2.2 kg/m^2^, AHI: 14.1 ± 6.6/h) into the SB + OSA group (RMMA index ≥2 times/h). In the SB + OSA group, 3 out of 10 had RMMA index ≥4 times/h, and mean the RMMA index was 4.7 times/h.

### 3.1. Demographic and sleep variables

Among 36 OSA patients, 10 had a RMMA index ≥2 times/h. In comparisons of demographic variables between 10 OSA patients with and 26 without SB, no significant differences were observed in demographic data, except for the frequency of self-awareness of tooth grinding (Table 1). Although the majority of sleep variables did not significantly differ between the two groups (Table 2), the percentage of REM sleep was significantly higher in OSA patients with than in those without SB (*p* = 0.02), as previous results on large study group (34). Regarding oromotor variables, the RMMA index was significantly higher in OSA patients with than in those without SB (*p* < 0.01), while the NSMA index did not significantly differ. In assessments of oromotor variables in the different sleep stages, the RMMA index in Stages N1 and N2 and Stage REM was significantly higher in OSA patients with than in those without SB (*p* < 0.01). The NSMA index was also significantly higher in OSA patients with than in those without SB for REM sleep (*p* < 0.01). This result is in agreement with recently published study (35). The episode type of RMMA was 1.3/h (0.5–5.7) for mixed events, 1.6/h (1.0–6.8) for phasic events, and 0.2/h (0–0.4) for tonic events in the OSA with SB group, and 0/h (0–0.6), 0/h (0–1.1), and 0/h (0–0.7) in the OSA without SB group, respectively. Although AHI did not significantly differ between the two groups, it was significantly lower in OSA patients with than in those without SB during REM sleep (*p* = 0.01). When REM-related phenotype of OSA was defined with a criteria of AHI-REM/AHI-NREM ratio ≥2 and AHI-NREM <15 events/h, none of the patients in the in OSA with SB group, and 3 of 26 patients in the in OSA without SB group fulfilled the definition. More OSA patients with SB (70%) had a low ArTH than those without SB (57.7%). Minimum SaO_2_ levels did not significantly differ between the two groups, while ArTH was significantly lower in OSA patients with than in those without SB (*p* = 0.04).

**Table 1:** Subject characteristics.

	all	0 ≤ RMMA<2	2 < RMMA	*p*
N	36	26	10	
Sex	F:7; M:29	F:6; M:20	F:1; M:9	0.65
Age (year)	49.6 ± 14.2	49.7 ± 14.1	49.3 ± 15.3	0.94
BMI (kg/m^2^)	24.5 ± 3.4	24.3 ± 3.8	25.0 ± 2.2	0.55
Existence of the attrition of teeth.	(−):29; (+):7	(−):20; (+):6	(−):9; (+):1	0.65
Awareness of teeth grinding during sleep	(−):25; (+):11	(−):21; (+):5	(−):4; (+):6	0.04*
Morning stiffness	(−):29; (+):7	(−):22; (+):4	(−):7; (+):3	0.37

Data are shown as the mean ± SD. RMMA: rhythmic masticatory muscle activity.

* Fisher’s exact test.

**Table 2:** Comparison of sleep variables and oromotor events in subjects with and without SB in the OSA group.

Group (number)	All (N = 33)	OSA (N = 26)	OSA + SB (N = 10)	*p*
Sleep period time (min)	441.3 ± 49.4	445.4 ± 42.8	430.7 ± 65.0	0.43
Sleep latency (min)	22.4 ± 44.9	15.6 ± 21.1	40.3 ± 15.5	0.14
Sleep efficiency (%)	78.9 ± 14.8	78.7 ± 15.0	79.5 ± 15.1	0.88
Sleep stages /SPT	-	-	-	
WASO (%)	16.6 ± 12.4	17.8 ± 14.1	13.3 ± 4.9	0.33
Stage N1 (%)	18.2 ± 10.2	19.0 ± 11.3	16.3 ± 7.0	0.50
Stage N2 (%)	45.4 ± 11.4	44.4 ± 12.0	47.7 ± 9.5	0.45
Stage N3 (%)	4.2 ± 6.1	4.6 ± 7.0	3.1 ± 3.1	0.52
Stage REM (%)	15.6 ± 6.2	14.1 ± 5.6	19.6 ± 6.3	0.02^#^
Arousal index (/h)	16.5 ± 14.0	18.4 ± 15.8	11.6 ± 4.8	0.19
Awakenings index (/h)	6.8 ± 2.8	6.9 ± 2.9	6.6 ± 2.5	0.81
RMMA index (/h)	0.6 (0–10.9)	0.1 (0–1.6)	3.3 (2.1–10.9)	<0.01*
Stage N1 + N2	0.5 (0–9.1)	0.1 (0–1.4)	2.8 (1.3–9.1)	<0.01*
Stage N3	0 (0–0.3)	0 (0–0.3)	0 (0–0.3)	0.33
Stage REM	0 (0–1.9)	0 (0–0.3)	0.1 (0–1.9)	<0.01*
NSMA index (/h)	4.8 (1.6–49.5)	4.9 (1.6–49.6)	3.8 (2.5–18.2)	0.70
Stage N1 + N2	6.6 (2.0–66.5)	6.4 (2.0–66.5)	7.1 (2.3–22.3)	0.83
Stage N3	0 (0–15)	0 (0–7.4)	0 (0–15)	0.67
Stage REM	2.8 (0–25.3)	2.8 (0–25.3)	3.4 (0–17.3)	<0.01*
AHI (/h)	17.5 (5.0–97.0)	25.0 (5.0–97.0)	12.9 (6.1–25.3)	0.06
Stage NREM	19.8 (1.0–100.3)	22.8 (1.0–100.3)	17.9 (2.0–27.4)	0.14
Stage REM	19.7 (0–66.0)	29.0 (0.8–66.0)	3.45(0–31.3)	0.01*
Lowest SpO_2_ (%)	83 (63–92)	83 (63–91)	85.5 (77–92)	0.14
Arousal threshold (cmH_2_O)	−14.6 (−35.3- -4.9)	−16.1 (−35.3- -4.8)	−11.0 (−17.1- -6.0)	0.04*

#*t*-test, *the Mann–Whitney *U*-test. SPT, sleep period time; WASO, wake time after sleep onset; RMMA, rhythmic masticatory muscle activity; NSMA, non-specific masticatory muscle activity; AHI, apnea-hypopnea index.

### 3.2. Temporal relationship between oromotor and respiratory events

**Figure 1:**
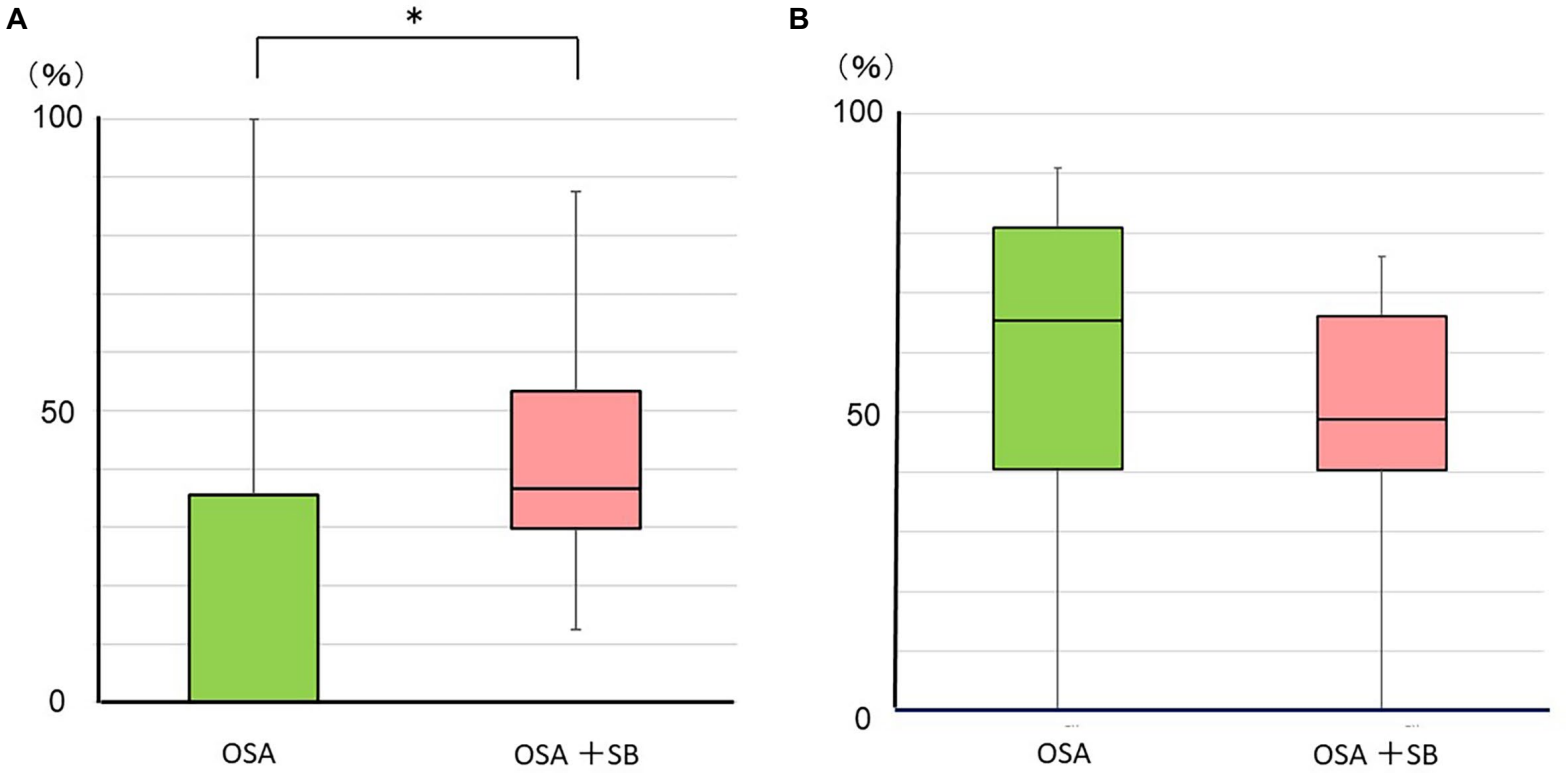
Temporal relationship between oromotor and respiratory events. **(A)** The percentage of RMMA following respiratory events. A significantly higher percentage of RMMA was scored during the 10-s period after respiratory events in the OSA + SB group (*p < 0.01: the Mann–Whitney *U*-test). **(B)** The percentage of NSMA following respiratory events. OSA: obstructive sleep apnea without sleep bruxism group. OSA + SB: obstructive sleep apnea with sleep bruxism group.

In the OSA group, 0% (0–35.5%) of total RMMA occurred during the 10-s period after respiratory events, whereas a significantly higher percentage of RMMA (36.6% [29.7–53.3%]) was detected during this period in the OSA + SB group (*p* < 0.01). However, 65.3% (40.4–80.9%) of NSMA events occurred in the 10-s period after respiratory events in the OSA group, whereas 48.8% (40.3–66.1%) occurred in this period in the OSA + SB group, with no significant differences between the two groups (Figure 1).

### 3.3. Relationships between oromotor and respiratory events

**Figure 2:**
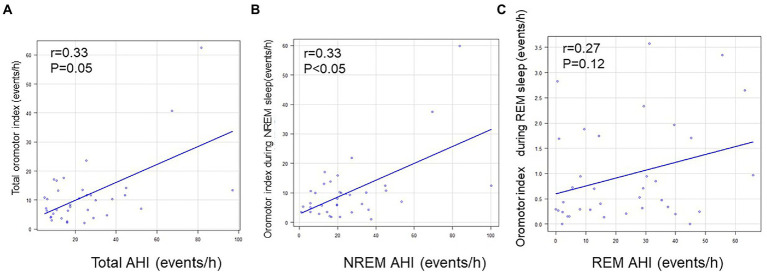
Relationships between AHI and the oromotor index. **(A)** Relationship between AHI and the oromotor index for the whole night. **(B)** Relationship between AHI and the oromotor index during NREM sleep. **(C)** Relationship between AHI and the oromotor index during REM sleep. The frequency of oromotor events positively correlated with that of apnea/hypopnea for the whole night and during NREM sleep (Spearman’s correlation coefficient). AHI: apnea-hypopnea index.

Spearman’s correlation analysis revealed a correlation between AHI and the oromotor index (the RMMA index and NSMA index) for the whole night (*p* = 0.05; *R* = 0.3) and during NREM sleep (*p* = 0.05; *R* = 0.3), but not during REM sleep (Figure 2).

**Figure 3:**
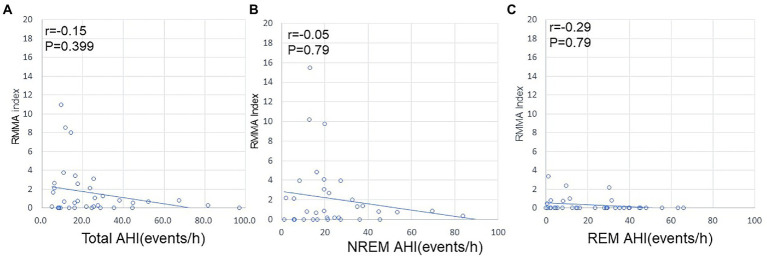
Relationships between AHI and the RMMA index. **(A)** Relationship between AHI and the RMMA index for the whole night. **(B)** Relationship between AHI and the RMMA index during NREM sleep. **(C)** Relationship between AHI and the RMMA index during REM sleep. No correlation was observed between AHI and the RMMA index.

**Figure 4:**
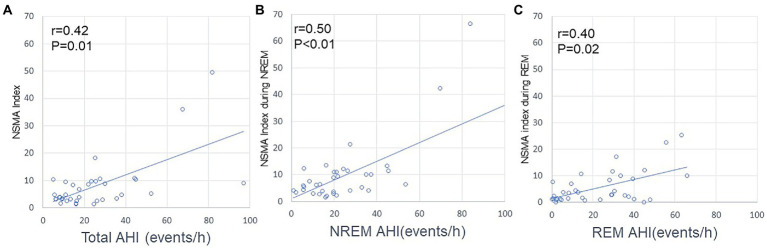
Relationships between AHI and the NSMA index. **(A)** Relationship between AHI and the NSMA index for the whole night. **(B)** Relationship between AHI and the NSMA index during NREM sleep. **(C)** Relationship between AHI and the NSMA index during REM sleep. The frequency of NSMA events positively correlated with that of apnea/hypopnea (Spearman’s correlation coefficient).

Regarding the RMMA index, no correlation was noted with AHI for the whole night, during NREM sleep, or during REM sleep (Figure 3). In contrast, a positive correlation was observed between AHI and the NSMA index for the whole night (*p* = 0.01; *R* = 0.416), during NREM sleep (*p* < 0.01; *R* = 0.5), and during REM sleep (*p* = 0.02; *R* = 0.4) (Figure 4).

### 3.4. Relationships between oromotor and arousal events

**Figure 5:**
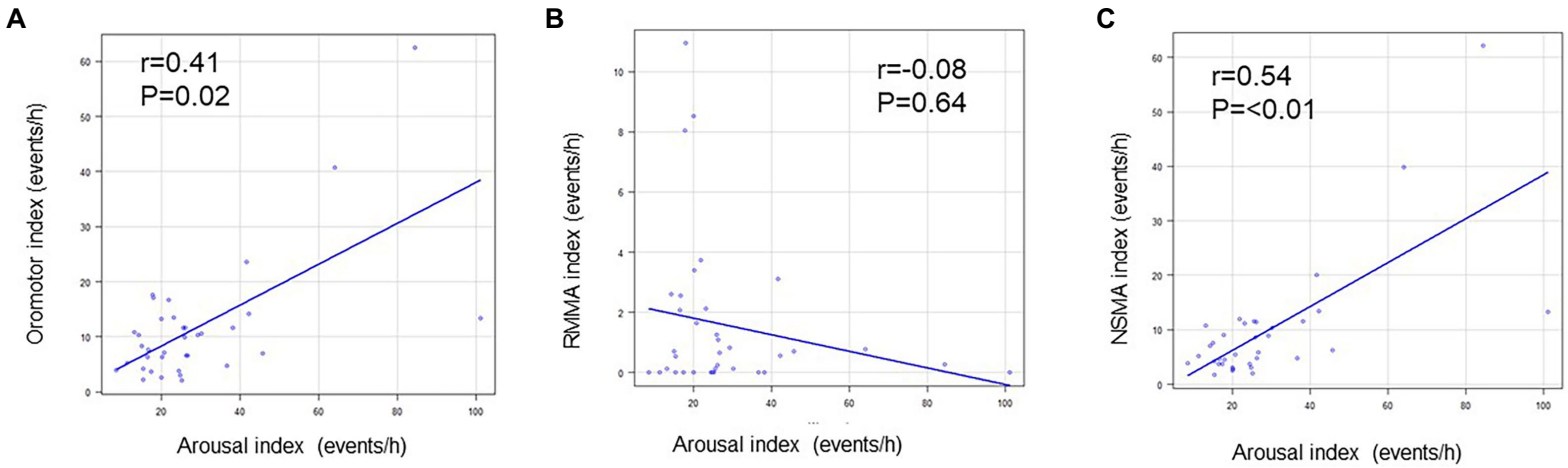
Relationships between arousal and oromotor events. **(A)** Relationship between the arousal index and oromotor index. **(B)** Relationship between the arousal index and the RMMA index. **(C)** Relationship between the arousal index and NSMA index. The arousal index positively correlated with the oromotor index and NSMA index (Spearman’s correlation coefficient).

The arousal index did not correlate with the RMMA index for the whole night, whereas it positively correlated with the NSMA index for the whole night (*p* < 0.01; *R* = 0.5). The frequency of oromotor events (the RMMA index and NSMA index) positively correlated with the arousal index for the whole night (*p* = 0.02; *R* = 0.4) (Figure 5).

## 4. Discussion

The present study investigated whether sleep variables and jaw motor responses were influenced by respiratory events in OSA patients with or without SB and examined the relationship between SB and OSA in OSA patients in relation to sleep stage dominancy. In the study population, 27.7% of OSA subjects were diagnosed with SB. OSA patients with SB showed NREM sleep dominancy for respiratory events with a lower arousal threshold. RMMA following respiratory events was more frequent in OSA patients with SB, while the frequency of NSMA following respiratory events did not significantly differ between the two groups. Even though the occurrence of RMMA did not correlate with AHI, the occurrence of NSMA positively correlated with AHI. These results suggest that OSA patients with SB have a unique physiological phenotype of OSA, and support previous findings showing that respiratory events were not a direct trigger for RMMA.

### 4.1. Prevalence of SB in OSA

Previous studies reported that the prevalence of SB varied in OSA patients; however, few objectively diagnosed SB (i.e., masticatory muscle EMG activity) in these patients. The SB subjects with an RMMA index of ≥4 times/h, was diagnosed in 33% (49/147) (10) to 47.8% (32/67) of OSA patients with a very high AHI (31.8 + 21.7 times/h) (13). The prevalence of SB was higher in other studies in which SB with an RMMA index of >2 times/h, was used as the cut-off value: it ranged between 53.7% (11) and 73.2% (30/41) in patients with moderate OSA (16.2 + 16.3 to 23.3 + 20 times/h) (12). Therefore, estimates of the prevalence of SB in OSA patients increased when a lower cut-off value was used for the diagnosis of SB. In the present study on moderate OSA patients (AHI = 17.5), the percentage of mild SB (27.7%) was lower than in previous studies using a diagnostic cut-off for the RMMA index of >2 times/h. Discrepancies in the findings of previous studies on the prevalence of SB in OSA patients may be attributed to the sample size and diversity of sample characteristics (i.e., obesity, ethnicity, a range of AHI, and the sleep architecture). Therefore, the present results need to be carefully interpreted due to the potential sampling bias associated with a small sample size consisting of heterogenic levels of OSA.

### 4.2. Sleep and respiratory variables

Consistent with previous findings on moderate to severe OSA (10, 12, 13), no significant differences were observed in sleep stages or arousal events between OSA patients with and without SB in the present study. Therefore, sleep was fragmented less by RMMA in the study population in the present study than in the studies described above. The larger amount of REM sleep in OSA patients with SB may be attributed to less frequent respiratory events during REM sleep because AHI during REM sleep was 8-fold lower in OSA patients with than in those without SB. These results suggest that OSA in this study population may be characterized by the NREM dominant type in OSA patients with SB (33, 36). Furthermore, the present study is the first to show that ArTH, another factor characterizing the phenotype of OSA, was significantly lower in OSA patients with than in those without SB. This is consistent with a recent finding on the role of a low ArTH in the pathophysiology of sleep-related movement disorders in OSA patients (23). Further studies are needed to clarify whether a lower ArTH, which potentially exacerbates the instability of arousal and respiration (31), is attributed to RMMA in OSA patients. Emerging evidence on OSA indicates that there are various phenotypes in the pathophysiology of OSA patients (37). Therefore, NREM dominancy and a low ArTH in OSA patients with SB suggest the importance of investigating the relationship between OSA and SB in order to obtain a more detailed understanding of these phenotypic differences.

In the present study, only 36.6% (median) of RMMA in OSA patients with SB occurred after respiratory events. NSMA was more frequent after respiratory events in OSA patients with (48.8%) and without (65.3%) SB. In addition, the correlation analysis revealed discrepancies in the relationship with arousal and respiratory events between RMMA and NSMA. A positive correlation was observed between AHI and the oromotor index (i.e., sum of NSMA and RMMA indexes). However, the NSMA index, rather than the RMMA index, positively correlated with AHI and the arousal index. These results support previous findings showing that NSMA occurred in association with a transient arousal response after respiratory events (17). In addition, the present results are supported by those in the previous studies in which RMMA and other nonspecific or tonic oromotor activities were scored separately in relation to respiratory events (12, 38). However, caution is needed because the occurrence of NSMA is more likely to be dependent on arousal events than on respiratory events (17, 39); 30–50% of NSMA did not follow respiratory events even though a positive correlation was observed between NSMA and respiratory events.

Although the occurrence of RMMA was previously associated with transient arousals (40), a correlation was not observed between the RMMA index and AHI/arousal events. Since OSA patients with SB had a lower AHI and ArTH, RMMA may be more responsive to mild rather than severe airway obstruction (i.e., less oxygen desaturation and respiratory flow limitations) (19). Transient arousal events related to NREM-REM sleep cycles were found to be important for the genesis of RMMA (29, 41). Therefore, RMMA may be more responsive to endogenously generated rather than respiratory-induced transient arousal events. Furthermore, in OSA patients, the protective role of the restoration of compromised airway patency is more likely to be achieved by NSMA than by RMMA because NSMA is associated with a more intense arousal response to severe oxygen desaturation (17, 39). These interpretations are supported by the findings of a recent clinical trial showing that a mandibular advancement device reduced the frequency of NSMA following respiratory events in OSA patients (39). Further studies are needed to investigate how variations in the responsiveness of RMMA and NSMA to respiratory events are associated with the characteristics of respiratory events (i.e., a decrease in SaO_2_ levels, apnea or hypopnea, the intensity of post-respiratory arousal events, and sleep-stage dominancy).

## 5. Study limitations

There are several limitations to interpreting the results of this study. First, this study was conducted with a small sample. A sample size of this study gave a statistical power of 74% to detect differences in mean of 28.1/h and 9.6/h for AHI during REM, assuming a common SD of 19.1/h, using a two-group t-test with a two-sided significance level of *p* < 0.05, between OSA without SB and OSA with SB group. Similarly, in the mean of 17.1 and 0% for the percentage of RMMA following respiratory events in each group, assuming a common SD of 11.3%, the study gave the power of 98%. Despite the small sample size, the statistical results can be considered reasonably reliable in understanding the findings. Second, pathophysiological phenotypes of OSA are very diverse. We cannot deny the possibility that OSA phenotypes can be heterogenous or biased with a small sample size. Although this study analyzed arousal threshold as one of physiological factors for defining the phenotypes, other physiological factors (loop gain etc.,) and the anatomical factors (e.g., craniofacial morphology) as well as the effect of body position (i.e., position related OSA) (15) were not considered for the analysis. In addition, the severity of most patients in this study were mild to moderate levels. Third, PSG was performed for one night. Previous studies have shown that there is a first-night effects on the sleep architecture (42) as well as the occurrence of RMMA (28): RMMA was significantly lower in the first night than the second night. Therefore, the RMMA in this study can be underestimated due to the first night effect. Thus, further large-sample studies are needed to clarify the physiological association between RMMA and respiratory events with a comparison among pathophysiological phenotypes.

## 6. Conclusion

The present study demonstrated that OSA patients with SB showed NREM sleep dominancy for respiratory events and a lower ArTH. In addition, NSMA, rather than RMMA, correlated with AHI and the arousal index. Within the limitations of the present study, namely, the small number of mild to moderate OSA patients examined, the results obtained suggest that OSA patients with SB exhibited more distinct phenotypes of the OSA pathophysiology than those without SB. The present results also indicate that RMMA and NSMA have a distinct relationship with respiratory events because RMMA was not directly triggered by respiratory events. Therefore, the comorbidity of OSA and SB may be diverse and clinicians need to be aware of heterogeneity in the treatment responses of OSA and/or SB in OSA patients when prescribing oral appliances.

## Data availability statement

The raw data supporting the conclusions of this article will be made available by the authors, without undue reservation.

## Ethics statement

The studies involving human participants were reviewed and approved by the Research Ethics Committee of Osaka University Graduate School of Dentistry, Osaka University Dental Hospital and Osaka Kaisei Hospital. The patients/participants provided their written informed consent to participate in this study.

## Author contributions

TK: conception and design. MuO, HM, HS, MoO, and MT: acquisition of the data. MuO, TK, MoM, HM, and HS: analysis, and interpretation of the data, responsible for all parts of the work, and ensuring that any concerns about its accuracy or integrity are properly examined and addressed. MuO and TK: drafting and revising the paper critically for important intellectual content. All authors contributed to the article and approved the submitted version.

## Funding

This study was supported by the Japan Society of the Promotion for Science (JSPS) #17K19753 and partially by the JSPS #22K1037, and by the funds from the Intractable Oral Disease at Osaka University Graduate School of Dentistry.

## Conflict of interest

The authors declare that the research was conducted in the absence of any commercial or financial relationships that could be construed as a potential conflict of interest.

## Publisher’s note

All claims expressed in this article are solely those of the authors and do not necessarily represent those of their affiliated organizations, or those of the publisher, the editors and the reviewers. Any product that may be evaluated in this article, or claim that may be made by its manufacturer, is not guaranteed or endorsed by the publisher.
